# Pathology of rituximab-induced Kaposi sarcoma flare

**DOI:** 10.1186/1472-6890-8-7

**Published:** 2008-07-23

**Authors:** Liron Pantanowitz, Klaus Früh, Sharon Marconi, Ashlee V Moses, Bruce J Dezube

**Affiliations:** 1 Department of Pathology, Baystate Medical Center, Tufts University School of Medicine, Springfield, MA, USA; 2 Vaccine and Gene Therapy Institute, Oregon Health and Science University, Portland, OR, USA; 3 Departments of Medicine (Hematology-Oncology Division), Beth Israel Deaconess Medical Center, Harvard Medical School, Boston, MA, USA

## Abstract

**Background:**

Kaposi sarcoma (KS) flare may occur following therapy with corticosteroids, as part of the immune reconstitution inflammatory syndrome seen with highly active antiretroviral therapy (HAART), and after rituximab therapy. The exact mechanism responsible for iatrogenic KS flare is unclear.

**Methods:**

A case of AIDS-associated cutaneous KS flare following rituximab therapy was compared to similar controls by means of immunohistochemistry using vascular makers (CD34, CD31), monoclonal antibodies to Human Herpesvirus 8 (HHV8) gene products (LNA-1, K5), as well as B-lymphocyte (CD20) and T-lymphocyte (CD3, CD4, CD8) markers.

**Results:**

CD20+ B-cell depletion with rituximab in KS flare occurred concomitantly with activation of the HHV8 immediate early gene protein K5. KS flare in this patient was successfully treated with liposomal doxorubicin and valganciclovir.

**Conclusion:**

Rituximab-induced KS flare appears to be related to HHV8 activation. Effective management of iatrogenic KS flare therefore depends upon the control of HHV8 viremia in conjunction with specific chemotherapy for KS.

## Background

Kaposi sarcoma (KS) is a multifocal, angioproliferative tumor related to Kaposi's Sarcoma Herpesvirus/Human Herpesvirus-8 (KSHV/HHV8) infection. HHV8 is a member of the gamma-herpesvirinae sub-family, in which gene expression and viral replication are regulated by latent, immediate early, early, and late viral gene transcription [1]. During latency, certain viral genes such as the Latency-associated nuclear antigen (LANA or ORF73) are critical for replication and maintenance of the HHV8 episome in latently infected cells. Immediate early genes (e.g. K3 and K5) code for transcriptional activators and are critical for initiating viral transcription [2]. K5, also known as modulator of immune recognition 2 (MIR2), is a transmembrane-spanning ubiquitin-ligase that mediates the ubiquitination of cytoplasmic proteins of other transmembrane proteins, such as major histocompatibility complex-I (MHC-I) molecules. Down regulation of MHC-I molecules by K5 gene expression during reactivation is important for evading the host's immune system, because this process inhibits the presentation of viral antigen to cytotoxic T cells [2].

Histologically, KS lesions are comprised of spindled shaped tumor cells, abnormal vessels and a variable chronic lymphoplasmacytic inflammatory infiltrate. KS lesions grow from an early patch stage, to form plaques, which ultimately develop into a nodular (tumor) stage. Most of the cells found in both KS lesions and HHV8-infected cultures are latently infected [3], although both lytic and latent phases of the HHV8 life cycle play significant roles in the pathogenesis of KS. In only a small percentage of infected cells is lytic replication of HHV8 observed [3].

KS exacerbation (flare or recrudescence) has been documented following therapy with corticosteroids [4-6], as part of the immune reconstitution inflammatory syndrome (IRIS) seen with highly active antiretroviral therapy (HAART) in HIV-infected persons [7-9], and after treatment with rituximab [10-13]. Rituximab is a chimeric murine/human immunoglobulin G anti-CD20 monoclonal antibody that mediates cytotoxicity against CD20+ B-cells. In the setting of human immunodeficiency virus (HIV) infection, rituximab has been increasingly used to treat multicentric Castleman disease (MCD). In a recent uncontrolled phase II study involving 21 patients with MCD that were treated with rituximab, 4 of the 11 patients (36%) who had cutaneous KS experienced progression of their KS lesions [13]. The exact mechanism(s) responsible for KS flare are unclear, and likely involve HHV8 activation and/or alteration of the host immune system.

We present, for the first time, the pathology of rituximab-induced KS flare. By means of immunohistochemistry, we set out to study the potential role of host immunity, as well as that of specific HHV8 gene products, in the pathogenesis of KS flare.

## Methods

Institutional Review Board approval from Baystate Medical Center and Beth Israel Deaconess Medical Center was obtained for this study. Informed consent was obtained from the patient to perform this study and publish the findings.

### Clinical case features

**Figure 1:**
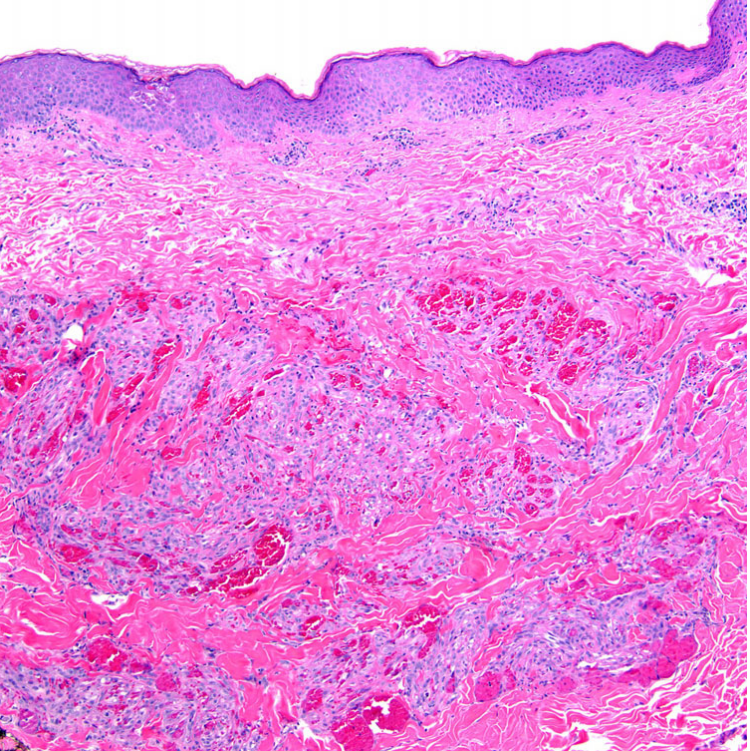
Cutaneous Kaposi sarcoma lesion, plaque stage (H&E stain; original magnification ×100).

**Figure 2:**
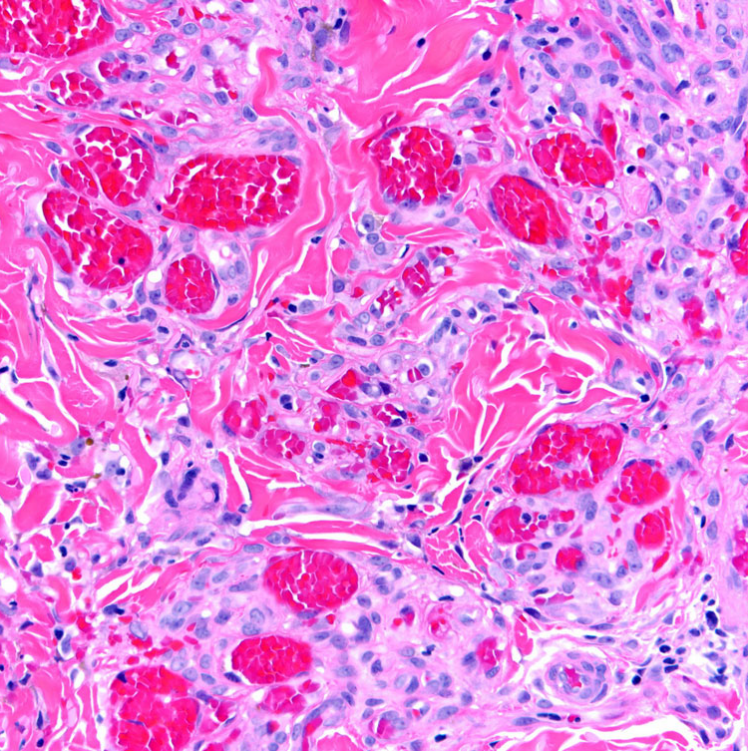
Higher power magnification of Kaposi sarcoma lesion showing infiltrating plump spindled tumor cells admixed with blood vessels and a sparse mononuclear inflammatory infiltrate (H&E stain; original magnification ×400).

A 36-year-old homosexual man known to be HIV-positive for two years presented with fever (104°F), chills, drenching night sweats, lassitude, diffuse myalgias and arthralgias. He had been started on HAART several months prior to this presentation, which was discontinued due to the development of a rash. Prior to the initiation of HAART, his CD4 T-cell count was 363 cells/mm^3 ^and his HIV viral load was greater than 100,000 copies/ml. His physical examination was notable for massive lymphadenopathy involving cervical, occipital, supraclavicular, axillary, groin and epitrochlear areas with lymph nodes measuring up to 1 cm in diameter. A CT scan also demonstrated marked lymphadenopathy of his chest, abdomen, and pelvis. Biopsy of an axillary node confirmed MCD, containing HHV8 (LNA-1) immunoreactive lymphoid cells. Treatment was begun with prednisone 1 mg/kg to control his symptoms, and rituximab 375 mg/m^2 ^once weekly for 4 consecutive weeks as definitive treatment for MCD. One week after starting rituximab, he developed a biopsy-proven KS plaque lesion on his leg (Figure 1 and 2), and another on his back. The patient continued his treatment of rituximab for MCD, was restarted on HAART for HIV infection, began liposomal doxorubicin for KS, and started daily valganciclovir for his HHV8 infection. He had rapid and dramatic normalization of all abnormalities, including resolution of his lymphadenopathy and complete disappearance of KS lesions.

### Immunohistochemistry

The aforementioned patient's biopsy was compared to similar (control) cutaneous plaque-stage KS lesions from two AIDS patients with stable KS disease. Formalin fixed, paraffin embedded routinely processed tissue was studied. The antibodies and dilutions used are summarized in Table 1. K5 mouse monoclonal antibody (Ab13.c5) is not commercially available, and was raised in the laboratory of one the authors (KF) against the c-terminal recombinant fragment of K5. All primary antibodies were diluted in Dako antibody diluent. Heat induced epitope retrieval was performed in Biogenex Citra buffer (plus buffer for LNA-1) for 10 minutes at 98°C. All immunohistochemistry was performed on a Dako Autostainer using a Signet Acuity kit (from Covance). H2O2 was used after antigen retrieval to block endogenous peroxidases for all antibodies except CD4 and CD8, in order not to completely inhibit staining in these two primary antibodies. All slides were then counter stained with hematoxylin. The immunoreactivity between our patient's KS flare lesion and the controls were compared.

**Table 1:** List of antibodies used for immunohistochemistry of Kaposi sarcoma.

**Antibody**	**Intended use**	**Source**	**Dilution**
CD34	Endothelial marker	Dako M765 Mouse monoclonal	1:500

CD31	Endothelial marker	Dako M0823 Mouse monoclonal	1:200

LNA-1	Latent HHV8 infection	Vector VP-H913 Rabbit polyclonal	1:40

K5	Lytic HHV8 infection	Ab13.c5 Mouse monoclonal	1:1000

CD3	T-lymphocytes	Dako A0452 Rabbit polyclonal	1:200

CD4	Helper T-cell subset	Vector VP-C318 Mouse monoclonal	1:20

CD8	Cytotoxic T-cell subset	Vector VP-C324 Mouse monoclonal	1:40

CD20	B-lymphocytes	Dako M0755 Mouse monoclonal	1:3000

## Results

**Figure 3:**
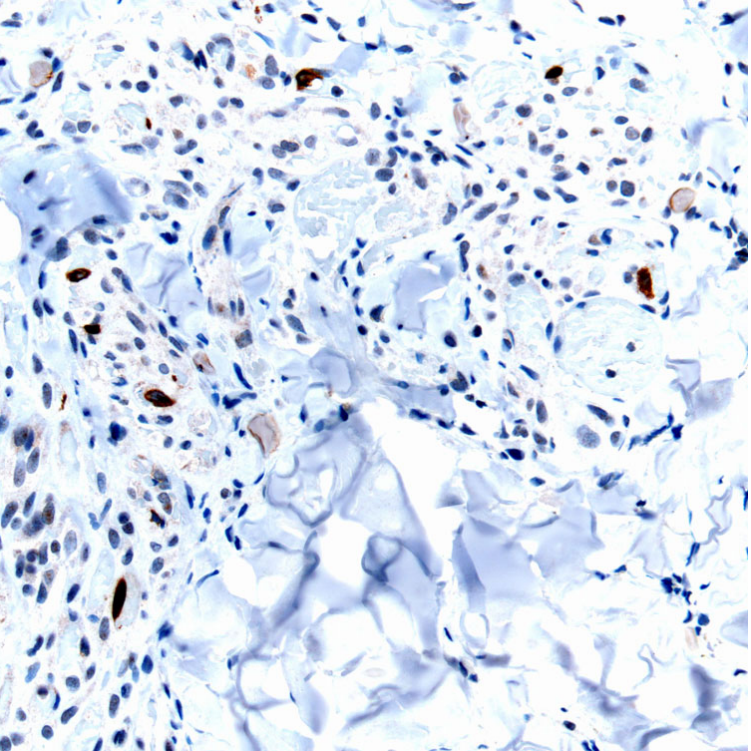
K5 immunoreactivity is shown in occasional tumor cells (arrows) in this KS flare lesion (K5 immunohistochemical stain; original magnification ×400).

**Figure 4:**
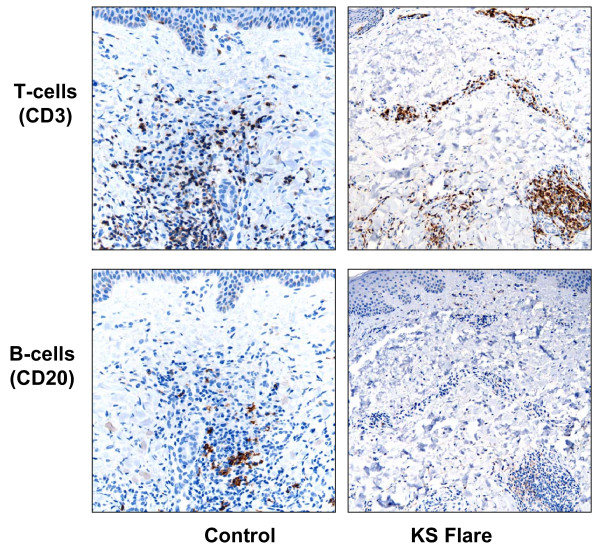
Comparison of the inflammatory component between control (left) and KS flare (right) specimens. Both lesions exhibit admixed T-cells (CD3), whereas only the KS flare tumor has a complete absence of B-cells (CD20).

The histomorphology of the KS flare case and that of the controls was unremarkable. In all specimens, CD34 and CD31 similarly highlighted spindled tumor cells and the abnormal vasculature within these lesions. LNA-1 nuclear granular immunoreactivity was of equal intensity and proportion in scattered KS tumor cells of all specimens. However, K5 staining was identified only in occasional cells (Figure 3) from the patient with KS flare. In the two control cases, both CD3 and CD20 cells were present, with a preponderance of T-lymphocytes identified. In the KS flare specimen, there were only T-cells present with a notable absence of B-lymphocytes (Figure 4). In all three cases T-cell subsets showed CD8 > CD4.

## Discussion

KS flare and/or exacerbation is a relatively newly described phenomenon related to the use of corticosteroids, HAART, and/or rituximab therapy. Although our patient received a combination of these drugs, his KS flare was temporally associated most closely with rituximab therapy. Moreover, he did not have other clinical features to support IRIS, such as a marked increase in CD4+ lymphocyte count and decreased plasma HIV-1 viral load after receiving HAART. While the initiation of HAART is most often associated with KS regression, there have certainly been accounts of KS flare following antiretroviral treatment. In one series involving nine patients with IRIS, the mean time to onset of KS flare was 5 weeks [9]. To the best of our knowledge, no studies have yet examined the actual pathogenesis of KS flare as a manifestation of IRIS.

To date, only a limited number of publications have reported KS flare and/or rapid progression in patients treated with rituximab [13]. One paper documented the brisk progression of classic KS in an 80-year-old man who was receiving prednisone and rituximab for severe autoimmune hemolytic anemia [12]. KS progression in this individual only occurred two weeks after completing rituximab infusions. His lesions did not regress, even months after stopping rituximab. Other investigators observed in responders to rituximab for HIV-associated MCD an aggravation of KS, even though the HIV-1 load and CD4 cell count in these individuals were unaffected [14].

The mechanism for KS development due to rituximab is undefined. Some authors postulate that by specifically depleting B cells, rituximab likely affects T-cell function (by eliminating B- and T-cell interactions), thereby facilitating KS development [12]. Other researchers found that clinical relapse of HIV-associated MCD in individuals who failed rituximab therapy was associated with increased blood HHV8 DNA levels and a worsening of KS [15]. In our study, we identified two unique findings in KS flare following rituximab; viz. the depletion of all lesional B lymphocytes and concomitant expression of the HHV8 gene product K5.

Lymphocytes are an integral component of KS tumors. Peripheral lymphocytes, particularly B-cells, are also the major latent reservoirs of HHV8. As compared to normal skin, KS tumors typically contain a high density of CD3+ T-lymphocytes, and fewer CD20+ B-lymphocytes [16]. In fact, the vast majority of KS infiltrating lymphocytes are of CD3+ CD8+ immunophenotype [17]. The evolution of KS from early to advanced disease is associated with a gradual decrease in the number of total and B lymphocytes. This is supported by a study involving 68 patients with classic KS, in which investigators found a statistically significant reduction in B-lymphocyte counts in individuals with advanced-stage KS (79/microL) compared with those in earlier stages of the disease (224/microL) [18]. Nadir B-cell counts in a similar study of HIV infected persons also documented an association with an increased risk of KS development [19]. Higher B-cell counts in this study of HIV-infected persons appeared to actually have a protective effect against the formation of KS lesions [19]. Therefore, it is easy to appreciate why B-cell depletion due to rituximab therapy in our case resulted in unwanted KS flare.

Exactly how B-cell depletion causes KS growth is most likely related to HHV8. Rituximab has previously been noted to cause Hepatitis B reactivation with related fulminant hepatitis [20]. In vivo, HHV8 within KS exists in a predominantly latent state, with less than 5% of infected cells expressing discernible lytic gene products. After stimulation, HHV8 can enter the lytic cycle by a reactivation process. Increased expression of the immediate early gene product K5 in KS flare tumor cells identified in our study may indicate HHV8 activation. In general, K5 is more frequently identified within advanced KS tumors compared to early KS lesions (unpublished observation). This explains why K5 was not identified in our control specimens of plaque stage. K5 is a modulator of immune recognition (ubiquitin ligase) that helps HHV8 evade the host's immune system during lytic replication [21].

## Conclusion

Our data demonstrate that B-cell depletion with rituximab in patient's at risk for KS development may activate HHV8, resulting in unwanted KS flare. Given the expanding therapeutic use of rituximab, caution is required when using this monoclonal antibody in patients at risk for developing KS. Nevertheless, in HHV8+ patients with MCD we feel that rituximab is still the treatment of choice. Finally, based upon the resolution of our patient's KS flare following appropriate therapy, effective management of iatrogenic KS flare appears to depend upon the control of HHV8 viremia in conjunction with chemotherapy for KS. Valganciclovir, when administered for 8 weeks, has been demonstrated in a randomized placebo-controlled trial to suppress HHV8 replication [22]. Although short term use of valganciclovir in patients who test positive for HHV8 could be considered, its clinical benefit has not been proven. Further work is required to better understand the interaction between B-cells and the lymphotropic HHV8.

## Competing interests

The authors declare that they have no competing interests.

## Authors' contributions

All authors contributed equally to the manuscript. LP coordinated the study and interpreted all immunostains. KF and AVM provided monoclonal antibodies. SM performed all stains. BJD was the treating physician. All authors read and approved the final manuscript.

## Pre-publication history

The pre-publication history for this paper can be accessed here:



## References

[B1] Ishido S, Wang C, Lee BS, Cohen GB, Jung JU (2000). Downregulation of major histocompatibility complex class I molecules by Kaposi's sarcoma-associated herpesvirus K3 and K5 proteins. J Virol.

[B2] Lacoste V, de la Fuente C, Kashanchi F, Pumfery A (2004). Kaposi's sarcoma-associated herpesvirus immediate early gene activity. Front Biosci.

[B3] Douglas JL, Gustin JK, Dezube B, Pantanowitz JL, Moses AV (2007). Kaposi's sarcoma: a model of both malignancy and chronic inflammation. Panminerva Med.

[B4] Gill PS, Loureiro C, Bernstein-Singer M, Rarick MU, Sattler F, Levine AM (1989). Clinical effect of glucocorticoids on Kaposi sarcoma related to the acquired immunodeficiency syndrome (AIDS). Ann Intern Med.

[B5] Joo M, Soon Lee S, Jin Park H, Shin HS (2006). Iatrogenic Kaposi's sarcoma following steroid therapy for nonspecific interstitial pneumonia with HHV-8 genotyping. Pathol Res Pract.

[B6] Jinno S, Goshima C (2008). Progression of Kaposi sarcoma associated with iatrogenic Cushing syndrome in a person with HIV/AIDS. AIDS Read.

[B7] Bower M, Nelson M, Young AM, Thirlwell C, Newsom-Davis T, Mandalia S, Dhillon T, Holmes P, Gazzard BG, Stebbing J (2005). Immune reconstitution inflammatory syndrome associated with Kaposi's sarcoma. J Clin Oncol.

[B8] Aboulafia DM (2005). Kaposi sarcoma flares during effective antiretroviral treatment. AIDS Read.

[B9] Leidner RS, Aboulafia DM (2005). Recrudescent Kaposi's sarcoma after initiation of HAART: A manifestation of immune reconstitution syndrome. AIDS Patient Care and STDs.

[B10] Casquero A, Barroso A, Fernández Guerrero ML, Górgolas M (2006). Use of rituximab as a salvage therapy for HIV-associated multicentric Castleman disease. Ann Hematol.

[B11] Gérard L, Bérezné A, Galicier L, Meignin V, Obadia M, De Castro N, Jacomet C, Verdon R, Madelaine-Chambrin I, Boulanger E, Chevret S, Agbalika F, Oksenhendler E (2007). Prospective study of rituximab in chemotherapy-dependent human immunodeficiency virus associated multicentric Castleman's disease: ANRS 117 CastlemaB Trial. J Clin Oncol.

[B12] Clifford KS, Demierre MF (2005). Progression of classic Kaposi's sarcoma with rituximab. J Am Acad Dermatol.

[B13] Bower M, Powles T, Williams S, Davis TN, Atkins M, Montoto S, Orkin C, Webb A, Fisher M, Nelson M, Gazzard B, Stebbing J, Kelleher P (2007). Brief communication: rituximab in HIV-associated multicentric Castleman disease. Ann Intern Med.

[B14] Marcelin AG, Aaron L, Mateus C, Gyan E, Gorin I, Viard JP, Calvez V, Dupin N (2003). Rituximab therapy for HIV-associated Castleman disease. Blood.

[B15] Neuville S, Agbalika F, Rabian C, Brière J, Molina JM (2005). Failure of rituximab in human immunodeficiency virus-associated multicentric Castleman disease. Am J Hematol.

[B16] Husein MR (2008). Immunohistological evaluation of immune cell infiltrate in cutaneous Kaposi's sarcoma. Cell Biol Int.

[B17] Valcuende-Cavero F, Febrer-Bosch MI, Castells-Rodellas A (1994). Langerhans' cells and lymphocytic infiltrate in AIDS-associated Kaposi's sarcoma. An immunohistochemical study. Acta Derm Venereol.

[B18] Stratigos AJ, Malanos D, Touloumi G, Antoniou A, Potouridou I, Polydorou D, Katsambas AD, Whitby D, Mueller N, Stratigos JD, Hatzakis A (2005). Association of clinical progression in classic Kaposi's sarcoma with reduction of peripheral B lymphocytes and partial increase in serum immune activation markers. Arch Dermatol.

[B19] Stebbing J, Gazzard B, Newsom-Davis T, Nelson M, Patterson S, Gotch F, Mandalia S, Bower M (2004). Nadir B cell counts are significantly correlated with the risk of Kaposi's sarcoma. Int J Cancer.

[B20] Scheinfeld N (2006). A review of rituximab in cutaneous medicine. Dermatol Online J.

[B21] Tomescu C, Law WK, Kedes DH (2003). Surface downregulation of major histocompatibility complex class I, PE-CAM, and ICAM-1 following *de novo* infection of endothelial cells with Kaposi's sarcoma-associated herpesvirus. J Virol.

[B22] Casper C, Krantz EM, Corey L, Kuntz SR, Wang J, Selke S, Hamilton S, Huang ML, Wald A (2008). Valganciclovir for suppression of human herpesvirus-8 replication: a randomized, double-blind, placebo-controlled, crossover trial. J Infect Dis.

